# Performance of a Machine Learning Algorithm to Predict Hypotension in Spontaneously Breathing Non-Ventilated Post-Anesthesia and ICU Patients

**DOI:** 10.3390/jpm14020210

**Published:** 2024-02-15

**Authors:** Johan T. M. Tol, Lotte E. Terwindt, Santino R. Rellum, Marije Wijnberge, Björn J. P. van der Ster, Eline Kho, Markus W. Hollmann, Alexander P. J. Vlaar, Denise P. Veelo, Jimmy Schenk

**Affiliations:** 1 Department of Anesthesiology, University of Amsterdam, Amsterdam UMC, Meibergdreef 9, 1105AZ Amsterdam, The Netherlands; j.tol2@amsterdamumc.nl (J.T.M.T.); j.schenk@amsterdamumc.nl (J.S.); 2 Department of Intensive Care Medicine, University of Amsterdam, Amsterdam UMC, Meibergdreef 9, 1105AZ Amsterdam, The Netherlands; 3 Laboratory of Experimental Intensive Care and Anesthesiology, Academic Medical Center Location, University of Amsterdam, Amsterdam UMC, Meibergdreef 9, 1105AZ Amsterdam, The Netherlands; 4 Department of Epidemiology and Data Science, University of Amsterdam, Amsterdam UMC, Meibergdreef 9, 1105AZ Amsterdam, The Netherlands

**Keywords:** hypotension, prediction, machine learning, ICU, PACU, critical illness

## Abstract

*Background*: Hypotension is common in the post-anesthesia care unit (PACU) and intensive care unit (ICU), and is associated with adverse patient outcomes. The Hypotension Prediction Index (HPI) algorithm has been shown to accurately predict hypotension in mechanically ventilated patients in the OR and ICU and to reduce intraoperative hypotension (IOH). Since positive pressure ventilation significantly affects patient hemodynamics, we performed this validation study to examine the performance of the HPI algorithm in a non-ventilated PACU and ICU population. *Materials & Methods:* The performance of the HPI algorithm was assessed using prospectively collected blood pressure (BP) and HPI data from a PACU and a mixed ICU population. Recordings with sufficient time (≥3 h) spent without mechanical ventilation were selected using data from the electronic medical record. All HPI values were evaluated for sensitivity, specificity, predictive value, and time-to-event, and a receiver operating characteristic (ROC) curve was constructed. *Results:* BP and HPI data from 282 patients were eligible for analysis, of which 242 (86%) were ICU patients. The mean age (standard deviation) was 63 (13.5) years, and 186 (66%) of the patients were male. Overall, the HPI predicted hypotension accurately, with an area under the ROC curve of 0.94. The most used HPI threshold cutoff in research and clinical use, 85, showed a sensitivity of 1.00, specificity of 0.79, median time-to-event of 160 s [60–380], PPV of 0.85, and NPV of 1.00. *Conclusion*: The absence of positive pressure ventilation and the influence thereof on patient hemodynamics does not negatively affect the performance of the HPI algorithm in predicting hypotension in the PACU and ICU. Future research should evaluate the feasibility and influence on hypotension and outcomes following HPI implementation in non-ventilated patients at risk of hypotension.

## 1. Introduction

Hypotension is common in the intensive care unit (ICU) [1], intra-operative [2] and post-operative environments [3,4]. The prevention of hypotension in all three environments may reduce the incidence of major adverse events, such as acute kidney injury (AKI), myocardial injury and death [5,6,7]. To facilitate a more proactive and timely treatment of hypotension, clinicians may benefit from incorporating machine learning and other advanced predictive algorithms into their decision-making process. One of these algorithms, the ‘Hypotension Prediction Index’ (HPI), has been shown to help clinicians reduce the incidence, severity, and duration of hypotension in the intraoperative period [8,9,10]. 

The HPI algorithm was developed to predict hypotension in the intraoperative period and in the ICU, and attempts to offer a real-time prediction of hypotension, defined as a mean arterial pressure (MAP) of <65 mmHg, based on the arterial pressure waveform [11]. The algorithm was originally developed using a mixed population consisting of ICU and surgical patients [11], and externally validated in mechanically ventilated surgical patients in the operating room (OR) and ICU [11,12,13]. The majority (60%) of their training and internal validation cohort was derived from ICU patients, but the proportion of mechanically ventilated patients in this cohort was not reported. In the only external validation of HPI in spontaneously breathing patients, carried out in patients undergoing cesarean delivery with spinal anesthesia, a similarly high sensitivity and specificity was found to the sensitivity and specificity in mechanically ventilated patients [11,12,13,14]. However, the hemodynamic profile of patients undergoing spinal anesthesia may not reflect that of patients recovering from general anesthesia, nor that of spontaneously breathing patients in the general ICU population.

Since prediction of hypotension by the HPI algorithm is based on “dynamic changes corresponding to physiologic interactions among left ventricular contractility, preload and afterload” [11], these changes may be substantially influenced by the changes in intrathoracic pressure and respiratory rate caused by mechanical ventilation [15,16]. We designed the present study to investigate the performance of HPI in a mixed population of spontaneously breathing critically ill and post-operative patients admitted to the Post Anesthesia Care Unit (PACU) or ICU, and to compare our results to those of validation studies performed earlier in mechanically ventilated patients.

## 2. Method

### 2.1. Study Design

To answer our research question, we combined data from two previously conducted prospective observational studies. Data collection was identical and took place during the same time period in patients at risk of hypotension in the PACU and ICU of the Amsterdam University Medical Center, Amsterdam, the Netherlands. 

Both studies were approved by the local ethics committee and registered in trial registries (NCT03376347 and NTR7150). Written informed consent was obtained prior to inclusion in the HYPE trial. Due to the nature of the PHYSIC study, where study measurements were initiated as soon as possible after ICU admission, deferred consent was obtained, and all patients or families could opt out of study participation. If a patient was temporarily unable to make independent decisions due their medical condition, deferred consent was obtained from a legal representative or from the patient if mental competence was regained during hospitalization. Study measurements for both studies took place in 2018 and 2019. Both studies were conducted according to the Declaration of Helsinki and the ICH Harmonized Tripartite Guideline for Good Clinical Practice.

### 2.2. Study Participants & Procedures

Invasive arterial blood pressure data were obtained in the PACU during the single-center, prospective sub-study [17] of the HYPE trial [18] and the ICU during the single-center, prospective PHYSIC study [19]. Both studies were performed at the Amsterdam University Medical Center, Amsterdam, the Netherlands. Participation in studies was mutually exclusive, i.e., patients could not be included in both studies.

The parent HYPE trial [18] included adult (≥18 years) patients scheduled for non-cardiac surgery under general anesthesia with an indication for invasive arterial blood pressure (BP) monitoring. In the post-HYPE sub-study, no further trial-related interventions took place, and BP and HPI measurements were strictly observational [17]. The PHYSIC study was an observational study that included adult (≥18 years) patients admitted to the ICU with an indication for invasive arterial pressure monitoring and an expected ICU stay of at least 8 h [19].

### 2.3. Objectives

The primary objective of this study was to evaluate the performance of HPI in non-ventilated, spontaneously breathing PACU and ICU patients. Special attention was directed towards the threshold of 85, since HPI > 85 is frequently used in research and is the threshold at which clinicians are actively alerted of impending hypotension. 

### 2.4. Outcome

Sensitivity, specificity, positive predictive value (PPV), negative predictive value (NPV), and time-to-event were calculated for all HPI thresholds from 0 to 100 in increments of 5, after which a receiver operating characteristic (ROC) curve was constructed and the area under the ROC curve (AUROC) was calculated. AUROC above 0.7 was considered acceptable [20]. 

### 2.5. Study Procedures

Invasive arterial pressure and HPI values were obtained for all patients using a FloTrac/HemoSphere hemodynamic monitoring platform (Edwards Lifesciences, Irvine, CA, USA) and FloTrac/Acumen IQ pressure transducers (Edwards Lifesciences, Irvine, CA, USA). Pressure transducers were placed at the level of the right atrium and zeroed before the start of study measurements. After the HYPE trial intraoperative interventional phase, arterial pressure was recorded from arrival in the PACU until discharge to the hospital ward and/or removal of the arterial cannula; no trial-related interventions were performed after arrival to the PACU [17]. In the PHYSIC study, arterial pressure was recorded in 499 patients admitted to the ICU, until either the arterial cannula was removed, the patient was discharged to a lower level of care, or until a recording duration of 72 h. During all measurements, the hemodynamic study monitor was covered and did not influence treatment. For the present analysis, participants were included only if ≥3 h of uninterrupted hemodynamic data without (non-)invasive positive pressure ventilation were available. 

### 2.6. Data Processing and Statistical Analysis

To evaluate the predictive ability of HPI, we modified the forward validation analysis, as described by Wijnberge et al., by extending the evaluated HPI range to all available thresholds [8], identical to the analysis of Van der Ven et al. [12]. Here, we defined an HPI alert as the exceedance of the selected threshold value for ≥1 min, e.g., 85. We used each HPI alert as the starting point of a 20 min window, which was checked for hypotension, defined as a mean arterial pressure (MAP) < 65 mmHg for ≥1 min. If HPI did not reach a value greater than the selected threshold, it was considered a negative prediction, and the same procedure was carried out. Timeframes were labeled true positive (TP), true negative (TN), false positive (FP), or false negative (FN). When a timeframe was labeled, the time window was shifted 20 min forward to ensure each event was only included in the analysis once. If hypotension occurred, the first occurrence of a MAP ≥ 65 was considered the end of the event. The absence of hypotension was only counted once per 20 min window to prevent the skewing of the data towards true negatives (TN). This procedure was repeated for all HPI thresholds ranging from 0 to 100 in increments of 5.

In accordance with previous HPI algorithm validations [8,11,12], we censored hemodynamic data that had a high probability of being influenced by data artifacts or clinical interventions, such as the administration of a fluid bolus or vasopressors, since this could lead to erroneous labeling. We excluded data that were highly likely to be affected by hemodynamic interventions and/or artifacts, such as arterial line sampling, positional changes and/or administration of vasopressors. 

The Time Weighted Average (TWA) of hypotension was calculated for each patient by determining the area under threshold, i.e., MAP < 65 mmHg, divided by the duration of the measurement period [18,19]. 

Patient demographics, type of surgery, reason for ICU admission and other relevant variables were extracted from the electronic medical record. Continuous data were reported as mean and standard deviation (SD) when normally distributed, or median [25th–75th percentiles] if not. Categorical data were reported as n (%). 

Data processing and statistical analysis were performed in MATLAB (v2018b, MathWorks, Natick, MA, USA) and R version 4.2.1 (R Foundation for Statistical Computing, Vienna, Austria). 

## 3. Results

A total of 559 patients were included in the post-HYPE and PHYSIC trials, of which 282 (50.4%) had ≥3 h of BP and HPI data available while not on mechanical ventilation. The remaining 277 (49.6%) patients were excluded due to insufficient (<3 h) BP and HPI data without concurrent mechanical ventilation. In total, 3.130 h, or 563,400 twenty-second segments, were eligible for analysis. The median measurement duration was 11.3 h [7.0–14.8]. There were a total of 4019 hypotensive events for a total duration of 24,489 min. The median number of events per patient was 4 [0–25], with a median duration of 2.0 min [1.5–2.9 min] per event. The median cumulative duration of hypotension per patient was 60.7 min [18.1–189.8] for 10.4% [2.4–29.3] of the total monitoring time. The TWA of hypotension was 0.03 mmHg [0.00–0.22].

The mean age of all participants was 63 years (13.3) and 186 (66%) of all eligible participants were male. Of all participants, 242 (85.8%) were admitted to the ICU, of which 153 (63.2%) were admitted after cardiopulmonary surgery. Other patient characteristics and reasons for admission are reported in Table 1. Extensive characteristics for all patients are reported in Appendix A. 

Sensitivity, specificity, time-to-event, PPV, NPV, and their respective confidence intervals for HPI values between 0 and 100 are reported in Table 2 in 5-HPI-point increments. In our analysis, sensitivity and NPV remain at 1.00 for HPI thresholds 0 through 80, after which a slight decrease is seen to 0.95 for sensitivity and 0.98 for NPV at an HPI value of 95. In contrast, specificity and PPV showed a gradual increase, from a specificity of 0.00 and PPV of 0.71 when the alert threshold was set at an HPI of 0, to a specificity of 0.89 and PPV of 0.90 at an HPI threshold of 95. Time-to-event steadily decreased from a median of 180 s at an HPI threshold of 0 to a median of 140 s at a threshold of 95. 

**Figure 1:**
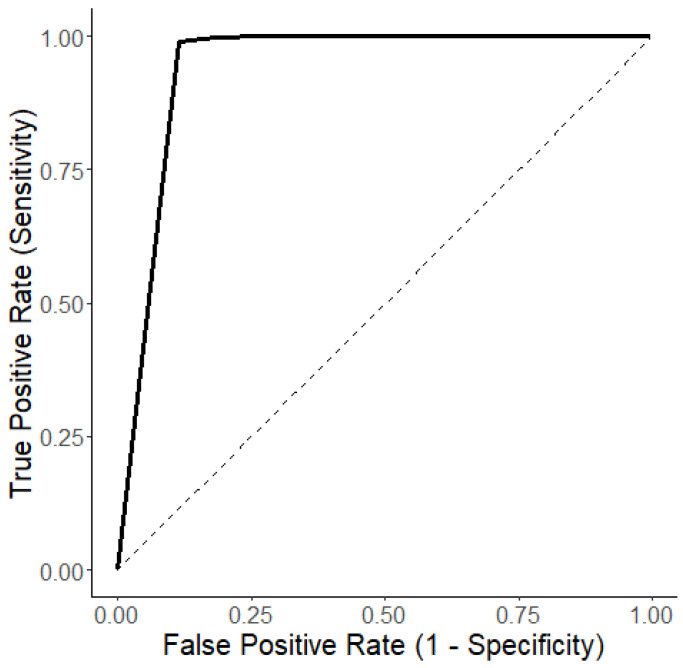
Receiver operating characteristic curve of the HPI for prediction of hypotension. AUROC of 0.94.

The most frequently used threshold to define an HPI ‘alert’ in clinical trials, HPI > 85, showed a sensitivity of 1.00, specificity of 0.79, median time-to-event of 160 s [60–378], PPV of 0.85, and NPV of 1.00. Figure 1 shows the ROC curve for all HPI thresholds and the corresponding AUROC of 0.94. An overview of all TN, TP, FN, and FP per threshold is included in Table S1 (Supplementary File).

## 4. Discussion

This study on the performance of HPI in 282 spontaneously breathing PACU and ICU patients shows that HPI holds excellent predictive value for hypotension, with a median time-to-event of 160 s at an HPI threshold of 85. Sensitivity, specificity, PPV, and NPV were similar to the results obtained in a study on HPI performance in mechanically ventilated patients [12], suggesting that physiologic changes due to the absence of positive pressure ventilation do not negatively affect the ability of HPI to predict hypotension. 

The HPI algorithm is currently a ‘black box’, using proprietary calculations with unknown variables, and was internally and externally validated in mechanically ventilated patients in the OR and ICU [11,12,13]. We hypothesized that the physiological interactions and hemodynamic changes HPI uses to predict hypotension [11] would be affected by both the difference in intrathoracic pressure and respiratory rate in spontaneously breathing patients compared to those on ventilatory support, altering the predictive ability of HPI. Hemodynamic variables such as pulse pressure variation and stroke volume variation—commonly used to evaluate patient hemodynamics—are known to be unreliable in spontaneously breathing patients [21]. Our results are similar to those of other studies validating HPI in mechanically ventilated patients [8,11,12,22], suggesting that the predictive ability of HPI is not affected by these major changes in respiratory status. This result is especially relevant in the early post-operative period and in the ICU, where patients are preferably not on respiratory support, while remaining at risk of hypotension [1,6,23]. Notably, hypotension in the first four days after surgery was significantly associated with a composite outcome of myocardial infarction and death, independently of preceding intraoperative hypotension [6]. Additionally, hypotension is a well-established risk factor for adverse outcomes, such as mortality and acute kidney injury, in the ICU [7]. In this study, patients spent 10.4% of the total monitoring time in hypotension, a substantial percentage for a patient population that may exhibit other warning signs of hypotension. A potentially substantial reduction in exposure to hypotension may be realized by employing hypotension prediction algorithms and similar early-warning systems to supplement the clinical decision-making process.

Multiple studies have shown that HPI reliably predicts hypotension before it occurs, in various patient populations and in various intraoperative and ICU settings [12,13,14]. As postoperative and ICU patients are generally diverse and preferably not on respiratory support, the validation of the HPI algorithm in non-mechanically ventilated patients was warranted, especially since there is major potential to reduce the time spent in hypotension in the post-operative and ICU setting. First, ICU practitioners estimate that their patients spend a median 15% of time each day in ICU in hypotension [1]. Secondly, post-operative hypotension appears more common and severe than previously thought [23]. This offers a possible explanation for the results of multiple clinical trials that successfully limited IOH without a significant positive effect on patient outcomes [24]. Thirdly, length of stay in the PACU and ICU is generally measured in hours to days, as opposed to minutes to hours intraoperatively, and thus the impact of preventing hypotension may be higher [1]. 

Future research should focus on HPI efficacy in preventing hypotension in non-ventilated patients at risk of hypotension.

## 5. Limitations

While the aforementioned ‘forward’ validation method was developed to provide clinically more meaningful results, validation of a predictive algorithm on a continuous scale remains complex and important caveats apply to its interpretation. For instance, when assessing the performance of HPI at a threshold of 10, all values above 10 will trigger an HPI alert. As a result, a nearly identical performance is observed for HPI alarms ranging from 0 to 70. As the HPI threshold rises, the alert range above the threshold decreases, and the performance statistics change accordingly. We believe this threshold-based approach most accurately reflects clinical practice. 

It was not feasible to continuously record hemodynamically relevant interventions during the study measurements due to the concurrent data collection for multiple patients. While our validation technique aims to censor data points that have a high likelihood of being influenced by clinical interventions or data artifacts, subtle changes preventing or causing hypotension may remain. This may have increased both the number of FP and FN HPI events, altering sensitivity and PPV of HPI. While the predictive ability of HPI does not suffer from the absence of mechanical ventilation, multiple secondary hemodynamic variables shown on the monitor screen, which can be used to guide hemodynamic interventions, are known to be unreliable in non-ventilated patients [21]. Clinicians aware of these caveats may abstain from using these variables and combine the HPI algorithm with other diagnostic means to prevent hypotension. The publication of the relevant hemodynamic features and interactions underlying the HPI algorithm would facilitate research into variables that remain valid and usable for guidance of hemodynamic treatment even after the cessation of mechanical ventilation. 

The TWA of hypotension in this study was relatively low compared to the TWA in both parent studies [18,19] and can be explained by the absence of general anesthesia and sedation in the majority of non-ventilated patients in the PACU and ICU. While this may have implications for the potential benefits gained by HPI-guided hemodynamic care, it does not hamper the evaluation of the ability of the HPI algorithm to predict hypotensive events, which is solely dependent on the incidence of hypotension and not its severity. 

## 6. Conclusions

This external validation of the HPI has shown that HPI reliably predicted hypotension defined as MAP < 65 mmHg in our mixed non-ventilated PACU and ICU cohort. As HPI is now validated in both mechanically ventilated and spontaneously breathing patients, future studies should evaluate the real-world performance of HPI in non-ventilated patients at risk of hypotension and the accompanying effect on patient morbidity and mortality. 

## Figures and Tables

**Table 1:** Patient characteristics.

Demographics	
*n*	282
ICU (%)	242 (85.8%)
Age (years)	62.7 (13.5)
Male (%)	186 (66.0%)
Weight (kg)	81.0 (18.1)
Length (cm)	174.4 (9.8)
BMI (kg/m^2^)	25.7 [23.6–29.0]
**Reason of Admission**	*n* (%)
Post-operative after cardiopulmonary surgery	153 (54.3)
Post-operative after major non-cardiopulmonary surgery	47 (16.7)
Sepsis	16 (5.7)
Subarachnoid hemorrhage	13 (4.6)
Neurologic with high ICP, NOS	12 (4.3)
Hypovolemic shock	8 (2.8)
Cardiac/cardiogenic shock	8 (2.8)
Respiratory failure, NOS	6 (2.1)
Distributive shock, NOS	4 (1.4)
Trauma	3 (1.1)
Spinal shock	3 (1.1)
Respiratory failure, pneumonia	3 (1.1)
OHCA	1 (0.4)
Other	4 (1.4)

Data presented as number (%), mean (standard deviation) or median [25th–75th percentiles]. ICP: intracranial pressure, ICU: intensive care unit, NOS: not otherwise specified, OHCA: out-of-hospital cardiac arrest.

**Table 2:** Impact of adjusting the HPI alert threshold on model performance.

HPI above Threshold ≥ 1 min	Sensitivity	Specificity	TTE (Seconds)	PPV	NPV
0	1.00	0.00	180 [80–400]	0.71	1.00
5	1.00	0.10	180 [80–400]	0.71	1.00
10	1.00	0.15	180 [60–400]	0.72	1.00
15	1.00	0.22	180 [60–400]	0.72	1.00
20	1.00	0.28	180 [80–400]	0.73	1.00
25	1.00	0.34	180 [80–400]	0.74	1.00
30	1.00	0.41	180 [80–400]	0.75	1.00
35	1.00	0.47	180 [60–400]	0.76	1.00
40	1.00	0.53	180 [80–400]	0.77	1.00
45	1.00	0.57	180 [60–400]	0.78	1.00
50	1.00	0.60	180 [60–400]	0.79	1.00
55	1.00	0.63	180 [60–400]	0.80	1.00
60	1.00	0.66	180 [60–400]	0.81	1.00
65	1.00	0.68	180 [60–400]	0.82	1.00
70	1.00	0.71	180 [60–380]	0.82	1.00
75	1.00	0.74	180 [60–380]	0.84	1.00
80	1.00	0.76	160 [60–380]	0.84	1.00
85	1.00	0.79	160 [60–375]	0.85	1.00
90	1.00	0.82	160 [60–360]	0.87	1.00
95	1.00	0.89	140 [60–340]	0.90	0.99
100	0.00	1.00	-	-	0.62

HPI: hypotension prediction index, TTE: time-to-event, PPV: positive predictive value, and NPV: negative predictive value.

## Data Availability

Data may be made available upon reasonable request.

## Supplementary Materials

The following supporting information can be downloaded at: https://www.mdpi.com/article/10.3390/jpm14020210/s1, Table S1: Matrix showing true positives, true negatives, false positives and false negatives for all HPI thresholds in the studies.

## Appendix A

**Table A1:** Characteristics of the included patients, split between PACU and ICU patients.

ICU	Overall	ICU Reason of Admission	*n* (%)
*n*	242	Post-operative after cardiopulmonary surgery	153 (63.2)
**Age**	62.4 (14.2)	Sepsis	16 (6.6)
**Male (%)**	165 (68.2)	Subarachnoid hemorrhage	13 (5.4)
**BMI** (kg/m^2^)	26.1 [23.8–29.1]	Neurologic with high ICP, NOS	12 (5.0)
**Shock type (if applicable) (%)**	*n* (%)	Hypovolemic shock	8 (3.3)
Distributive	29 (12.0)	Cardiac/cardiogenic shock	8 (3.3)
Cardiogenic	26 (10.7)	Post-operative after non-cardiopulmonary surgery	7 (2.9)
Mixed type	9 (3.7)	Respiratory failure, NOS	6 (2.5)
Hypovolemic	3 (1.2)	Distributive shock, NOS	4 (1.7)
Obstructive	1 (0.4)	Trauma	3 (1.2)
**SOFA**	9.0 [7.0–11.0]	Spinal shock	3 (1.2)
		Respiratory failure, pneumonia	3 (1.2)
		OHCA	1 (0.4)
		Other	4 (1.7)
**PACU**	**Overall**	**ASA Classification**	***n* (%)**
*n*	40	I	2 (5.0)
**Age (years)**	64.4 (8.4)	II	34 (85.0)
**Male (%)**	21 (52.5)	III	4 (10.0)
**Weight (kg)**	73.1 (15.37)	Surgery type	*n* (%)
**BMI** (kg/m^2^)	24.55 [21.6–26.3]	Gastro-Intestinal	37 (92.5)
**Duration of surgery (min.)**	262 [224–429]	Gynecological oncology	1 (2.5)
		Other	2 (5.0)

Data presented as number (%), mean (standard deviation) or median [25th–75th percentiles]. ASA: American society of anesthesiologists, SOFA: sequential organ failure assessment, ICP: intracranial pressure, ICU: intensive care unit, NOS: not otherwise specified, OHCA: out-of-hospital cardiac arrest, and PACU: post-anesthesia care unit.

## Abbreviations

AKI	Acute kidney injury
AUROC	Area under receiver operating characteristic curve
FN	False negative
FP	False positive
HPI	Hypotension Prediction Index
ICU	Intensive Care Unit
MAP	Mean arterial pressure
NPV	Negative predictive value
OR	Operating room
PACU	Post-anesthesia care unit
PPV	Positive predictive value
ROC	Receiver operating characteristic
SD	Standard deviation
TN	True negative
TP	True positive
TWA	Time-weighted average
