# Supplementary material for: Performance of a Machine Learning Algorithm to Predict Hypotension in Spontaneously Breathing Non-Ventilated Post-Anesthesia and ICU Patients
Source: J Pers Med. 2024 Feb 15;14(2):210. doi: 10.3390/jpm14020210 (PMC10890176; doi:10.3390/jpm14020210)
Supplement: Supplementary file 1 [file jpm-14-00210-s001.zip › jpm-2859281-supplementary.pdf]

Table S1. Matrix showing True Positives, True Negatives, False Positives and False Negatives for all studies HPI thresholds.

HPI above threshold ≥1 minute	True positives	False positives	False Negatives	True negatives
0	4892	1995	0	6
5	4893	1966	0	222
10	4890	1938	0	350
15	4894	1892	0	521
20	4900	1821	0	702
25	4896	1758	0	900
30	4887	1658	0	1138
35	4903	1558	0	1397
40	4910	1462	0	1645
45	4922	1382	0	1816
50	4928	1305	0	1967
55	4944	1261	0	2099
60	4933	1175	0	2259
65	4908	1114	0	2415
70	4887	1043	0	2564
75	4874	963	0	2701
80	4820	903	2	2850
85	4787	823	5	3001
90	4653	714	14	3193
95	4091	470	39	3624
100	0	0	2530	4203
